# Laparoscopic Cholecystectomy in Cirrhotic Patients: Feasibility in a Developing Country

**DOI:** 10.4103/1319-3767.39620

**Published:** 2008-04

**Authors:** Mohammad Tayeb, Muhammad R. Khan, Nazia Riaz

**Affiliations:** Department of Surgery, The Aga Khan University, Karachi, Pakistan

**Keywords:** Cirrhosis, laparoscopic cholecystectomy, outcome

## Abstract

**Background/Aims:**

Although laparoscopic cholecystectomy (LC) has become the procedure of choice for cholelithiasis in the general population, many consider cirrhosis as a relative or absolute contraindication for laparoscopic surgery. The aim of this study was to confirm the safety of LC in cirrhotic patients in our set-up.

**Materials and Methods:**

This is a retrospective case series including all the patients with cirrhosis who underwent LC for gallstones from January 2000 to December 2006 at our institution. Data were analyzed for Child class, indication for surgery, hospital stay, and procedure-related morbidity and mortality. Results are given as mean ± standard deviation.

**Results:**

Thirty patients, including 21 females (median age: 42 years) underwent LC during the study period. There was no operative mortality. Twenty-four patients belonged to Child class A and 6 belonged to Child class B. Mean operative time was 80 ± 26 min. There was no incidence of bile duct injury, but two patients (6.7%) required conversion to open procedure. Mean hospital stay was 3 ± 2.7 days. Postoperative morbidity was observed in seven patients, including postoperative deterioration of liver function in 2, worsening of ascites in 2 and pneumonia, and port-site infection in 1. Two patients had significant drop in hemoglobin requiring blood transfusion.

**Conclusions:**

Cirrhosis is not a contraindication for LC and it can be performed safely in compensated cirrhotic patients with acceptable morbidity and mortality.

Viral hepatitis B- and C-related chronic hepatitis is endemic in Pakistan, and chronic liver disease secondary to these infections is one of the major health problems in our country. A study conducted in Pakistan indicated that 4.3% of the population tested was sero-positive for hepatitis B surface antigen and 6% for hepatitis C antibody.[1] Gallstones occur twice as frequently in cirrhotic patients as they do in noncirrhotic patients.[2,3] This is possibly due to hemolysis, hypersplenism, reduction in biliary acidity, functional alterations in the gallbladder, and metabolic liver failure resulting in an increase in unconjugated bilirubin secretion.[4] With increasing prevalence of viral hepatitis and chronic liver disease, surgeons are now more frequently encountering cirrhotic patients with symptomatic gallstones requiring intervention.[5]

Open cholecystectomy (OC) performed in cirrhotic patients has been consistently associated with high morbidity and mortality.[2,6] The major causes of poor outcome associated with OC in these patients are intraoperative blood loss, ascites, wound and pulmonary infections, and gastrointestinal tract hemorrhage.[4,7] Although laparoscopic cholecystectomy (LC) has become the procedure of choice for cholelithiasis in the general population, many consider cirrhosis as a relative or absolute contraindication for laparoscopic surgery.[8,9] Several studies conducted in developed countries in the recent years have documented the safety and efficacy of LC in cirrhotic patients.[10,11] Comparative studies are also available that show that LC is more beneficial than OC in this group of patients.[12,13] The objective of our study was to assess the feasibility and safety of LC in cirrhotic patients at our institution with a specific view to analyze if results similar to published literature can be replicated in developing countries.

## MATERIALS AND METHODS

We retrospectively reviewed the medical records of all the patients with cirrhosis who underwent LC from January 2000 to December 2006 at our institution. Records were retrieved using the ICD-9-CM coding system. The diagnosis of cirrhosis was established by preoperative work-up, gross appearance of liver during surgery, or biopsy performed during surgery. The data were analyzed for patient's demographics, etiology of cirrhosis, Child class, indications for surgery, procedure performed, duration of surgery, procedure-related morbidity, and mortality and duration of hospital stay. The results are given as mean ± standard deviation.

Standard laparoscopic approach was adopted for LC. First port was introduced infraumbilically by means of the open (Hassan) technique. After inducing pneumoperitoneum using carbon dioxide, the rest of the three standard ports were introduced under vision. In two patients, an additional port was used to retract the liver. In three patients, a harmonic scalpel was used to dissect the gallbladder from the liver, while a diathermy spatula was used in majority of the patients. Six patients required drain placement peroperatively. The drains were removed within 24 h in four patients and within 4–6 days in the other two patients. Twenty-two patients received low-salt diet and diuretics and five patients received salt-free albumin postoperatively. Eight patients did not receive any special treatment.

## RESULTS

Thirty patients (21 females and 9 males) underwent LC during the study period. Median age of the patients was 42 years (range: 24–76 years). Eighteen patients had associated comorbid diseases including hypertension and diabetes mellitus. Eighteen patients were found to have hepatitis C, four had hepatitis B and two had co-infection with B and C, while six had cryptogenic cirrhosis with no evidence of infectious or metabolic abnormality. Eighteen patients had preoperative diagnosis of cirrhosis, while 12 patients were incidentally found to be cirrhotic on laparoscopy. After evaluation, 24 patients were classified as belonging to Child class A, and 6 as Child class B. Two patients were initially classified as Child class C, but they were optimized and down-staged to Child class B before surgery.

All the patients had symptomatic gallstones as shown in Table 1. The patients were classified according to the American Society of Anesthesiologists (ASA) classification, and six patients were labeled as ASA I, 18 as ASA II and the remaining 6 as ASA III. The details of the operative procedure are shown in Table 1. Four patients had laparoscopic partial cholecystectomy, while two patients (6.7%) required conversion to open procedure. The reasons for conversion were excessive bleeding in one case and obscure anatomy secondary to acute inflammation and adhesions in the other case. Mean operative time was 80 ± 26 min. Two patients required blood product transfusion during surgery.

**Table 1:** Details of indications, operative procedure, and morbidities related to the procedure

I. Indications for surgery	
Biliary colic	21
Acute cholecystitis	06
Gallstone pancreatitis	03
II. Procedure performed	
Laparoscopic cholecystectomy (LC)	18
Laparoscopic partial cholecystectomy	04
LC + liver biopsy	06
LC converted to OC	02 (6.7%)
III. Post-operative morbidities	
Total number of patients with complications	07 (23%)
Liver decompensation	02
Worsening of ascites	02
Pneumonia + port-site infection	01
Drop in hemoglobin requiring blood transfusion	02

There was no operative mortality. Postoperative morbidity was observed in seven (23%) patients as shown in Table 1. Two patients had postoperative deterioration of liver function resulting in progression from Child class A to Child class B. They required readmission due to liver decompensation (on the 5^th^ and 7^th^ postoperative days, respectively). Another two patients had worsening of ascites requiring diuretics and salt-free albumin. One patient developed postoperative pneumonia and port-site infection resulting in a prolonged hospital stay. Two patients had a significant drop in hemoglobin level requiring blood transfusion. All these complications were treated medically and the patients were discharged from the hospital in a healthy status. There was no bile duct injury and no patient required a re-visit to operating room for any complications. Mean hospital stay was 3 ± 2.7 days. Mean follow-up was only 2 months, although some patients were followed up to 4 years.

## DISCUSSION

Our institution is a tertiary care hospital with reasonable expertise in laparoscopic surgery. We perform about 300 LCs in one calendar year, but in keeping with the situation in the other developing countries, the facilities and expertise for advanced laparoscopy are still limited in our set-up. Previously, laparoscopic approach was considered a relative contraindication in cirrhotic patients at our hospital. With increasing experience in laparoscopic surgeries, LC has been attempted in cirrhotic patients and the results have been encouraging. Selection of patients according to liver reserve seems to be the key issue. Only patients classified as Child classes A and B underwent LC in our series. Two patients with Child C were initially managed conservatively and optimized to improve their liver function. Once their Child status improved, they were operated upon with good results. This finding is consistent with observations in the other published series, as Child class C has consistently been shown to have poor outcome.

Our results of LC in cirrhotics are comparable to the previously published reports and a comparison has been summarized in Table 2. In our study, hepatitis caused by B and C viruses was the leading cause of cirrhosis, but none of our patients had alcoholic liver disease as an underlying pathology. This is in contrast with the western literature, where alcoholic liver disease is frequently prevalent.[14,15] Forty percent of the patients in our study were incidentally diagnosed with cirrhosis during LC and it was confirmed by liver biopsy. The rate of incidental diagnosis in our study seems to be higher when compared to other reports published recently.[14–16] Although all these patients belonged to Child class A, we are now considering routine screening for hepatitis B and C in all surgical patients.

**Table 2:** Comparison of our results with other published reports

	Cucinotta *et al*.[14]	Poggio *et al*.[15]	Sleeman *et al*.[21]	Tayeb *et al*.
Total number of patients	22	26	25	30
Mean age (years)	58	51	53	42
Gender				
Males	9	15	8	9
Females	13	11	17	21
Etiology of cirrhosis			
Viral	18	12	18	24
Alcohol	03	06	04	00
Other	01	08	03	06
Diagnosis of cirrhosis				
Preoperative	18	24	19	18
Intraoperative	04	02	06	12
Child class					
A	12	22	16	24
B	10	04	09	06
C	00	00	00	00
Conversion to open	11%	12%	00%	6.7%
Mean operative time (minutes)	115	114	-	81
Mean hospital stay (days)	4	2.3	1.7	3
Complications	36%	19%	32%	23%
Mortality	00	00	00	00

Mean operative time in our series was 80 min, which is significantly shorter than that of reported in the earlier literature.[14,15] Our conversion rate to OC was 6.7%, which is comparable to that reported in other studies for cirrhotic patients.[14,15] For the general population, conversion rate during LC ranges from 0 to 9%.[17,18] Our rate of conversion to open procedure (6.7%) was similar to published data for LC conversion in a noncirrhotic patient population,[19,20] reflecting the fact that cirrhotic patients are not necessarily a high-risk group for conversion.

Two patients in our study required blood product transfusion including packed red cells, fresh frozen plasma, and platelets, due to significant drop in hemoglobin level. Both these patients had a platelet count of <100,000/mm^3^ and deranged coagulation manifested by an International Normalized Ratio (INR) of more than 1.5 preoperatively. Therefore, we suggest that not only the Child class, but also the complete coagulation profile including platelet count, prothrombin time, activated partial thromboplastin time and INR should be routinely assessed in these patients preoperatively and corrected if possible.

The complication rate of 23% in our study is comparable to 19% of Poggio,[15] 36% of Cucinotta,[14] and 32% of Sleeman series.[21] The main complications in our series were worsening of ascites and liver function, which were successfully managed conservatively. There was no bile duct injury or postoperative bleeding reported in our study.

The results of our study confirm that LC is a safe operative approach in most patients with Child class A and B cirrhosis and symptomatic cholelithiasis. The LC offers the advantages of reduced blood loss due to magnification, reduced wound-related complications, shorter anesthesia as well as surgical periods and reduced hospital stay. Additionally, an important advantage of this approach is the few number of right upper quadrant adhesions postoperatively. This will be an advantage for patients having a liver transplantation in future.[18,21] The use of LC for patients with hepatitis B- and C-related cirrhosis is advantageous for the surgical team because laparoscopy reduces the possibility of prick injuries with sharps.

In summary, our results show that LC in cirrhotic patients with well-compensated liver function (Child classes A and B) is a feasible option. Laparoscopic approach should be considered as the procedure of choice in this subgroup of patients with symptomatic cholelithiasis even in the developing countries, and especially those with reasonable expertise in laparoscopic surgery.
